# Risk factors for early mortality after AIDS in the cART era: A population-based cohort study in Italy

**DOI:** 10.1186/s12879-015-0960-6

**Published:** 2015-06-12

**Authors:** Barbara Suligoi, Antonella Zucchetto, Enrico Grande, Laura Camoni, Luigino Dal Maso, Luisa Frova, Saverio Virdone, Stefano Boros, Marilena Pappagallo, Martina Taborelli, Vincenza Regine, Paolo De Paoli, Diego Serraino

**Affiliations:** Centro Operativo AIDS, Istituto Superiore di Sanità, Rome, Italy; Unit of Epidemiology and Biostatistics, CRO Aviano National Cancer Institute, via Gallini 2, 33081 Aviano, Italy; Health and social care Section, National Institute of Statistics, Rome, Italy; Scientific Directorate, CRO Aviano National Cancer Institute, Aviano, Italy

**Keywords:** AIDS, Early mortality, Survival, Italy, cART

## Abstract

**Background:**

Despite the dramatically improved survival due to combination antiretroviral therapies (cART), life expectancy of people with HIV/AIDS remains lower than that of the general population. This study aimed to estimate, at a population level, the survival experience of Italian people with AIDS (PWA) and to quantify the prognostic role of selected factors at diagnosis in the risk of early mortality (i.e., within six months from AIDS diagnosis).

**Methods:**

A population-based, retrospective-cohort study was conducted among Italian PWA diagnosed between 1999 and 2009 and recorded in the national AIDS registry. The vital status, up to December 2010, of 14,552 PWA was ascertained through a record linkage procedure with the Italian mortality database. Survival probabilities were estimated through Kaplan-Meier method. To identify risk factors for early mortality from any cause, odds ratios (ORs) and corresponding 95 % confidence intervals (CIs), adjusted for major confounders, were computed using multivariate logistic regression models.

**Results:**

Of the 5,706 deaths registered among the 14,552 PWA included in the study, 2,757 (18.9 %) occurred within six months from AIDS diagnosis. The probability of surviving six months increased from 81.2 % in PWA diagnosed in 1999–2000 to 82.9 % in 2009, while the 5-year survival augmented from 60.7 % in PWA diagnosed in 1999–2000 to 65.4 % for PWA diagnosed in 2005–2006. Elevated risks of early mortality were associated to older age (OR = 5.28; 95 % CI: 4.41-6.32 for age ≥60 vs. <35 years), injecting drug use (OR = 1.71; 95 % CI: 1.53-1.91 vs. heterosexual intercourse), and CD4 count <50 cells/mm^3^ at AIDS diagnosis (OR = 1.87, 95 % CI: 1.55-2.27 vs. ≥350). Elevated ORs for early mortality also emerged for PWA diagnosed with primary brain lymphoma (OR = 11.66, 95 % CI: 7.32-18.57), or progressive multifocal leukoencephalopathy (OR = 4.21, 95 % CI: 3.37-5.27).

**Conclusions:**

Our study documented, among Italian PWA, the high - though slightly decreasing - frequency of early mortality in the full cART era. These findings indicate the need for enduring and ameliorating preventive actions aimed at timely HIV testing among all individuals at risk for HIV infection and/or those who present diseases known to be related with HIV infection.

## Background

Development of combination antiretroviral therapies (cART) in the mid-1990s revolutionized the care of HIV-infected patients, leading to a marked decline in morbidity and mortality associated with HIV infection [1]. Although the increasingly widespread use of cART has substantially improved the survival of HIV-infected people who have access to these drugs [2], at a population level the life expectancy of HIV-infected people is still lower than that observed in the general population [3].

The yearly number of new AIDS diagnoses recorded by the Italian national AIDS registry (RNAIDS) has steadily decreased in the last decade almost halving its total, i.e., from nearly 2,000 cases in 2000 to approximately 1,000 cases in 2011–2013 [4]*.* In Italy, cART for HIV-infected individuals is free of charge and administered according to regularly updated standardized guidelines [5]. Nonetheless, some factors, such as late HIV diagnosis, can delay access to cART and have an impact on life expectancy of AIDS patients [6]. A previous study on Italian people with AIDS (PWA) reported that mortality was particularly elevated, as compared to the general population, during the first year following AIDS diagnosis [7].

The present epidemiological study intended to estimate, at a population level, the survival of Italian PWA in the cART era and to quantify the prognostic role of selected factors at diagnosis on the risk of early mortality (i.e., herein defined as death within six months following AIDS diagnosis).

## Methods

A population-based, retrospective-cohort study was conducted among Italian PWA diagnosed between 1999 and 2009 and reported to the RNAIDS. In Italy, AIDS diagnoses (based on clinical criteria in accordance with the 1993 revised European AIDS definition [8]) are compulsorily reported to the RNAIDS, a national surveillance system previously described in detail [4]*.* For the present analysis, the following information collected at AIDS diagnosis by the RNAIDS was used: age, gender, area of residence, years of education, HIV transmission mode [mutually exclusive groups: injecting drug use (IDU), heterosexual intercourse, homosexual male intercourse], year of diagnosis, CD4 cell count, use of cART before AIDS, date of first HIV-positive test, and AIDS-defining diseases. AIDS-defining diseases were classified into: i) infectious diseases (*Pneumocystis carinii* pneumonia, tuberculosis, candidiasis, recurrent pneumonia, brain toxoplasmosis, other opportunistic infections); ii) neoplasms (invasive cervical cancer, Kaposi sarcoma, immunoblastic lymphoma, Burkitt lymphoma, primary brain lymphoma); and iii) other conditions (HIV encephalopathy, HIV wasting syndrome, and progressive multifocal leukoencephalopathy). Individuals with more than one AIDS-defining disease were assigned to the condition with the worst prognosis [7]*.*

The vital status of PWA (as of December 31, 2010) was provided by the Italian National Institute of Statistics (ISTAT), where death certificates from the whole national territory are centrally collected and electronically stored in the Italian mortality database. Death certificate registration is mandatory (with completeness being around 100 %) and since January 1999, the ISTAT database of death certificates is managed according to European guidelines [9–11]*,* and it includes names and surnames. In agreement with the national law regulating the use of sensible data, we carried out a record linkage between the RNAIDS and the Italian mortality database following the inclusion, in 2012, of this study in the National Statistical Plan and the consequent authorization of the Italian Data Protection Authority. The record linkage was performed using an upgraded version of Software for Automated Linkage in Italy (SALI), a software application developed and validated in Italy to identify the same individuals in two different databases ensuring anonymity through a blinded procedure [7, 12]*.*

Data regarding the 17,969 PWA reported to the RNAIDS from 1999 to 2009 were linked to data concerning the 6,895,720 deaths reported to the Italian mortality database between 1999 and 2010. The study period selected for the Italian mortality database was one-year longer than the RNAIDS one to allow for the detection of deaths for PWA diagnosed in 2009.

Specifically excluded from this analysis were: (1) PWA who were non-Italian citizens, to limit false-negative results of the linkage due to non-Italian names and surnames (*N* = 3,184); (2) PWA who at time of death resided or died in the provinces of Trento and Bolzano because names and surnames were not reported in death certificates (*N* = 167); 3) pediatric cases (i.e., aged less than 13 years at AIDS diagnosis) (*N* = 48); 4) PWA with coincident dates of AIDS diagnosis and death (*N* = 18). Therefore, 14,552 adult Italian PWA constituted the study group.

The main study outcome was death from any cause at six months following AIDS diagnosis (i.e., these were considered early mortality deaths), and it constituted the focus of this analysis. Two statistical approaches were used, a time-dependent, survival analysis and a logistic regression analysis. For the survival analysis, we considered failure events the deaths for any cause that occurred at 6 months, one, three, and five years after AIDS diagnosis. Survival time was computed as the time elapsed from the date of AIDS diagnosis to the date of death (for PWA identified in the Italian mortality database), to end of follow-up or to December 31, 2010 (i.e., censored cases) whichever came first*.* In addition to time trends in short-term survival (i.e., six months), we computed data also for one, three, and five years survival following AIDS diagnosis to document temporal changes in both short- and long-term mortality rates. The survival probability of PWA after AIDS diagnosis was estimated by means of the Kaplan–Meier method [13]. To identify factors reported at AIDS diagnosis potentially associated to early mortality, odds ratios (ORs) and 95 % confidence intervals (CIs) were computed using a multivariate logistic regression adjusted for variables that were statistically significant at univariate analysis [13]*.*

## Results

**Fig. 1:**
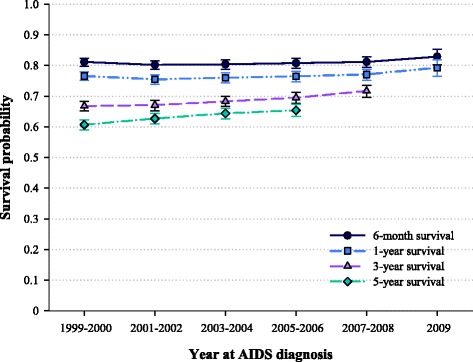
Six-month, one-, three-, and five-year survival probabilities of Italian people with AIDS by year at diagnosis. Italy, 1999–2009

Of the 14,552 PWA diagnosed between 1999 and 2009 included in this study, 5,706 were deceased as of December 31, 2010. Of these 5,706 deaths, 2,757 (18.9 % of all PWA) were early mortality deaths, i.e., they occurred within six months after AIDS diagnosis. Over time, the probability of surviving six months from AIDS diagnosis increased from 81.2 % for cases diagnosed in 1999–2000 to 82.9 % (+2.1 %) for those diagnosed in 2009. Improvements were also registered for three- and five-year survival (e.g., five-year survival increased from 60.7 % in PWA diagnosed in 1999-2000 to 65.4 % in those diagnosed in 2005–2006, +7.7 %) (Fig. 1).

Table 1 reports the distribution of PWA and the multivariate ORs for early mortality, according to selected variables collected at AIDS diagnosis. The risk of early mortality increased with age (OR = 5.28; 95 % CI: 4.41-6.32, for PWA aged ≥60 years vs. <35 years), and it was similar in males and females. Geographical and socioeconomic differences emerged, with PWA residing in northern or southern Italy at higher risk for early mortality than PWA in central Italy. Less educated PWA (i.e., those with 5 or less years of education) were at higher risk of death (OR = 1.25), and IDU showed a 71 % elevated risk (95 % CI: 1.53-1.91) of early mortality than heterosexuals. The risk of early mortality was lower (OR = 0.84, 95 % CI: 0.74-0.94) in PWA diagnosed in 2007–2009 vs. those diagnosed in 1999–2002. Conversely, an inverse association emerged between early mortality risk and CD4 cell count, particularly for PWA with a CD4 cell count lower than 50 CD4 cells/mm^3^ at AIDS diagnosis (OR = 1.87; 95 % CI: 1.55-2.27 vs. ≥350 CD4 cells/mm^3^). Use of cART before AIDS was recorded in 36.2 % of these PWA (OR = 1.12; 95 % CI: 1.02-1.23 vs. untreated).

**Table 1:** Distribution of AIDS cases by selected characteristics collected at AIDS diagnosis and odds ratios^a^ for early mortality. Italy, 1999-2009

	AIDS cases (No. = 14552)	Early mortality (No. = 2757)	
	No.	No. (%)	OR (95 % CI)
Age at AIDS diagnosis (yrs)			
13-34	2839	362 (12.8)	1^b^
35-39	3543	545 (15.4)	1.19 (1.03-1.37)
40-44	3305	597 (18.1)	1.48 (1.28-1.71)
45-49	1944	410 (21.1)	1.98 (1.69-2.32)
50-54	1104	253 (22.9)	2.38 (1.98-2.87)
55-59	764	181 (23.7)	2.59 (2.10-3.19)
≥60	1053	409 (38.8)	5.28 (4.41-6.32)
*χ* ^*2*^ for trend (*p-*value)			373.13 (*p* < 0.01)
Gender			
Male	11388	2203 (19.3)	1^b^
Female	3164	554 (17.5)	1.04 (0.93-1.16)
Area of residence^c^			
Centre	3481	594 (17.1)	1^b^
North	8043	1534 (19.1)	1.14 (1.02-1.27)
South	2836	594 (20.9)	1.35 (1.19-1.54)
Education (yrs)			
≥9	3563	563 (15.8)	1^d^
6-8	6603	1202 (18.2)	1.11 (0.99-1.24)
≤5	2127	519 (24.4)	1.25 (1.08-1.44)
Unknown	2259	473 (20.9)	1.23 (1.07-1.42)
*χ* ^*2*^ for trend (*p-*value)^d^			9.57 (*p* < 0.01)
HIV transmission mode^e^			
Heterosexual intercourse	5088	887 (17.4)	1^b^
Homosexual male intercourse	2862	472 (16.5)	1.04 (0.91-1.19)
Injecting drug use	5646	1147 (20.3)	1.71 (1.53-1.91)
Year at AIDS diagnosis			
1999-2002	6519	1255 (19.3)	1^b^
2003-2006	5084	965 (19.0)	0.92 (0.84-1.02)
2007-2009	2949	537 (18.2)	0.84 (0.74-0.94)
*χ* ^*2*^ for trend (*p-*value)			8.79 (*p* < 0.01)
CD4 cell count/mm^3^ at AIDS diagnosis^c^			
≥350	1135	140 (12.3)	1^b^
200-349	1623	286 (17.6)	1.45 (1.17-1.81)
50-199	5369	984 (18.3)	1.57 (1.29-1.90)
<50	5957	1187 (19.9)	1.87 (1.55-2.27)
*χ* ^*2*^ for trend (*p-*value)			46.80 (*p* < 0.01)
Use of pre-AIDS antiretroviral drugs^c^			
No	8834	1620 (18.3)	1^b^
Yes	5265	1005 (19.1)	1.12 (1.02-1.23)

^a^Odds ratios (OR) and 95 % confidence intervals (CI) estimated using multivariate logistic regression models adjusted for age, gender, area of residence, education, HIV transmission mode, year and CD4 cell count at AIDS diagnosis

^b^Reference category

^c^The sum does not add up to the total because of missing values

^d^Unknown excluded

^e^The sum does not add up to the total because of other, not considered, HIV transmission modes and missing values

**Fig. 2:**
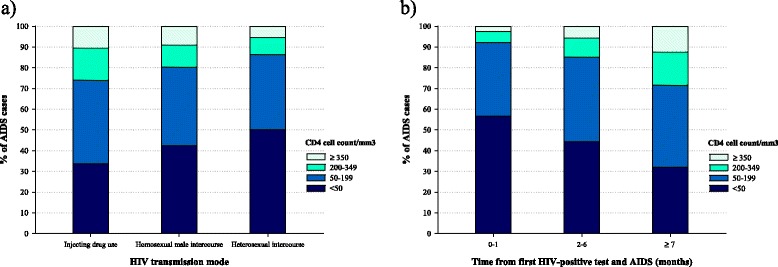
AIDS cases distribution* by CD4 cell count/mm^3^ at diagnosis according to: **a**. HIV transmission mode; and **b**. Time elapsed between first HIV-positive test and AIDS. Italy, 1999–2009, * 468 PWA with missing data on CD4 cell count were excluded

A CD4 count at AIDS diagnosis below 50 cells/mm^3^ was more frequently observed among PWA who acquired HIV infection through heterosexual intercourse (50.1 %), or homosexual male intercourse (42.3 %) than among injecting drug users (33.8 %) (Chi-square test, *p* < 0.01) (Fig. 2a). PWA who first tested HIV-positive one month before AIDS had a significantly higher proportion of low CD4 count (below 50 cells/mm^3^), as compared to PWA who first tested HIV-positive seven or more months before AIDS (56.7 % vs. 32.1 %, Chi-square test, *p* < 0.01) (Fig. 2b).

The risk of early mortality was not affected by time elapsed between first HIV-positive test and AIDS, even after stratification for cART before AIDS (e.g., OR = 1.17, 95 % CI 0.73-1.86, for cART treated, and OR = 1.01, 95 % CI 0.87-1.18, for untreated, 0–1 months vs. ≥13) (Table 2).

**Table 2:** Distribution of AIDS cases according to time from first HIV-positive test and use of antiretroviral drugs and odds ratios^a^ for early mortality. Italy, 1999-2009

	Use of pre-AIDS antiretroviral drugs^b^			
	Yes		No	
	Cases/deaths		Cases/deaths	
	No./No.	OR (95 % CI)	No./No.	OR (95 % CI)
Time from first HIV-positive test to AIDS diagnosis (months)^c^				
0-1	113/25	1.17 (0.73-1.86)	4723/889	1.01 (0.87-1.18)
2-3	136/20	0.75 (0.46-1.24)	861/152	0.94 (0.75-1.17)
4-6	105/22	1.14 (0.69-1.86)	168/27	0.82 (0.53-1.28)
7-12	112/16	0.76 (0.44-1.31)	122/23	1.06 (0.66-1.72)
≥13	4600/874	1^d^	2435/433	1^d^

^a^Odds ratios (OR) and 95 % confidence intervals (CI) estimated using multivariate logistic regression models adjusted for age, gender, area of residence, education, HIV transmission mode, and year at AIDS diagnosis

^b^Data on pre-AIDS use of antiretroviral drugs were not available for 337 cases

^c^Information on date at first HIV-positive test was missing for 840 cases

^d^Reference category

The number of defining conditions diagnosed at time of AIDS was a strong, statistically significant, prognostic factor for early mortality. PWA with three or more AIDS-defining diseases showed a 1.97-fold higher risk of early mortality, compared to those with one disease (Table 3). Elevated ORs for early mortality emerged, as compared to those diagnosed with *Pneumocystis carinii* pneumonia, for PWA diagnosed with non-Hodgkin lymphomas, particularly primary brain lymphoma (OR = 11.66, 95 % CI: 7.32-18.57), and progressive multifocal leukoencephalopathy (41.5 % of PWA with such disease died within six months from diagnosis) (OR = 4.21, 95 % CI: 3.37-5.27).

**Table 3:** Distribution of AIDS cases according to AIDS-defining diseases and odds ratios^a^ for early mortality. Italy, 1999-2009

	AIDS cases	Early mortality	
	No.	No. (%)	OR (95 % CI)
AIDS-defining diseases^b^			
*Infectious diseases*			
*Pneumocystis carinii* pneumonia	2650	393 (14.8)	1^c^
Tuberculosis	1089	126 (11.6)	0.81 (0.65-1.00)
Candidiasis	2943	404 (13.7)	0.84 (0.72-0.98)
Recurrent pneumonia	466	62 (13.3)	0.91 (0.68-1.23)
Other infections	1476	266 (18.0)	1.22 (1.02-1.45)
Brain toxoplasmosis	856	175 (20.4)	1.43 (1.16-1.75)
*Neoplasms*			
Invasive cervical cancer	104	9 (8.7)	0.59 (0.29-1.24)
Kaposi sarcoma	773	80 (10.4)	0.82 (0.63-1.07)
Immunoblastic lymphoma	707	234 (33.1)	3.18 (2.59-3.89)
Burkitt lymphoma	286	95 (33.2)	3.48 (2.62-4.62)
Primary brain lymphoma	85	54 (63.5)	11.66 (7.32-18.57)
Other conditions			
HIV encephalopathy	999	231 (23.1)	1.55 (1.28-1.88)
HIV wasting syndrome	1655	436 (26.3)	1.78 (1.52-2.09)
Progressive multifocal leukoencephalopathy	463	192 (41.5)	4.21 (3.37-5.27)
No. of AIDS-defining diseases at diagnosis			
1	11670	2048 (17.6)	1^c^
2	2261	510 (22.6)	1.27 (1.13-1.42)
≥3	621	199 (32.1)	1.97 (1.64-2.37)

^a^Odds ratios (OR) and 95 % confidence intervals (CI) estimated using multivariate logistic regression models adjusted for age, gender, area of residence, education, HIV transmission mode, year and CD4 cell count at AIDS diagnosis

^b^For patients with more than one AIDS-defining disease, only the most severe was considered

^c^Reference category

## Discussion

Our population-based investigation focused on early mortality of AIDS cases, and it showed that, over time, a slight amelioration in the proportion of PWA who died within six months from AIDS (from 81.2 % in PWA diagnosed in 1999–2000 to 82.9 % in 2009). Improvements were also documented in five-year survival, from 60.7 % in 1999–2002 to 65.4 % in 2005–2006. Our findings, together with the reduced risk of early mortality for PWA diagnosed in more recent years that emerged from the multivariate analysis, seemed to suggest a progress in the management of AIDS that has led to a slight, but clinically significant, improvement also in short term survival. This latter encouraging observation is consistent with findings reported elsewhere [14], and it probably reflects general improvements in the management of comorbidities and non-HIV specific interventions, in addition to enhancement of antiretroviral therapies [15].

Overall, the findings indicated that, 18.9 % of Italian PWA, diagnosed between 1999 and 2009, died within six months from AIDS diagnosis. This quantification of early mortality risk is particularly high when compared with findings from clinical trials [16]. It should be noted, however, that this is a population-based descriptive investigation, including 40.9 % of PWA with less than 50 CD4 cell count at AIDS diagnosis in contrast, for instance, with a median of 280 CD4 cell count at enrolment in the cited trial [16]. In addition, and in agreement with similar observations recorded in France [6] and in Brazil [17]*,* the results of this analysis strongly indicated that the largest part of one year post-AIDS mortality occurs within six months after diagnosis.

Approximately one fifth of Italian AIDS cases diagnosed between 1999 and 2009 were aged 50 years or older, and older age was confirmed to be a strong negative prognostic factor also for early mortality. In this study, the negative effect of older age on early mortality was clear overall, after adjusting by CD4 cell count, HIV transmission group, or other factors, or when stratifying by time elapsed from first HIV-positive test to AIDS. This finding is in agreement with the results of a recently published article showing that, in France, PWA aged 60 years or more were at a nearly 3-fold higher risk of late HIV presentation, and this event was strongly associated to an elevated risk of death in the first six months after AIDS diagnosis [18]*.*

Overall, women with AIDS, who constitute about 22 % of Italian PWA included in this analysis, showed a risk of early mortality similar to men. This result seems in line with lack of gender difference reported in the proportion of HIV late presenters in Italy [19]*,* or life expectancy of HIV-infected people on cART [14, 20]*.* It must be stressed that a strong HIV prevention effort has been addressed to women in recent years in Italy. National guidelines focusing on HIV testing in pregnancy have been issued to avoid mother-to-child transmission and, indirectly, to identify unaware HIV-infected women [21]*.*

Some of the characteristics herein investigated have been previously recognized as risk factors for mortality of PWA [7], namely injecting drug use, older age, a low number of CD4 cells at AIDS diagnosis, and severe AIDS-defining diseases. Nevertheless, some findings deserve to be highlighted.

In our study, the use of cART before AIDS was associated to a slight increased risk of early mortality (OR = 1.12). In Italy, cART is administered free of charge to every HIV-infected individual fitting standardized clinical and laboratory criteria [22]*.* In line with the literature, our results suggest that cART-treated individuals benefit from a longer AIDS-free lifetime and that they develop a full-blown AIDS when the immune suppression is already at an advanced stage. Therefore, the risk of early mortality in the pre-AIDS treated group was related to the final depletion of the immune system and/or the presence of other life threatening conditions. Moreover, this finding may also be due to channelling bias (i.e., treated people are those with the worse prognosis) and/or to the presence of unmeasured confounding factors.

The type of AIDS-defining disease turned out to be a strong negative prognostic factor for early mortality. Among these, non-Hodgkin lymphomas and multifocal leukoencephalopathy were associated to more than 3-fold higher ORs, similarly to what reported in other population-based and clinical studies conducted in several high-income countries [20, 23].

Finally, IDU showed the highest early mortality risk compared to PWA who acquired the infection through sexual contact; however, among IDU we observed the lowest proportion of individuals with a low CD4 cell count, suggesting that the elevated risk of early mortality was, in this group. mainly attributable to a longer duration of HV infection and/or to comorbidities, such as liver diseases or cancer, rather than to immune suppression.

The main strengths of this population-based, retrospective-cohort study are the national coverage for both AIDS notifications data and death certificates; the quality of data, in terms of completeness and representativeness; the large size of the study population; and the long observation period. It is relevant to mention that the linkage between the two independent databases allowed the detection of all PWA who died for any reason, and not only for AIDS-related conditions, without the inconvenience of losses to follow-up that can occur in cohort studies based on a unique data source. However, we are aware that prospective cohort studies, in comparison with retrospective ones could provide a larger set of information and a more precise assessment of follow-up time for the survival analysis.

Conversely, a major study limitation is the exclusion of non-Italian PWA from the analysis whose contribution to the AIDS epidemic in Italy is relevant. However, the linkage methodology used in this study –despite taking into account frequent spelling errors in names– could be less sensitive when matching foreign names and surnames due to a higher inaccuracy, in addition to a higher tendency of foreign citizens to migrate. Information on date of HIV-seroconversion is not collected by RNAIDS, as well as duration of adherence to treatment with cART; therefore, the duration of HIV infection of PWA and major parameters of treatment with cART could not be used for the aims of this investigation. Moreover, data on post-AIDS use of cART are not available at the RNAIDS, and this represents another important limitation that could have concealed the impact of cART on short term mortality.

## Conclusions

In the full cART era, population-based investigations focusing on the mortality of PWA and on prognostic factors represent valuable pieces of epidemiological evidence for clinicians and for researchers alike. These study results indicated that, in Italy, about one fifth of PWA died within six months after AIDS diagnosis, and this early mortality risk slightly decreased in the last decade. The main risk factors of early mortality were older age, use of injection drugs, a very low CD4 cell count and the presence of a number of life threatening conditions at AIDS diagnosis. These findings indicate the need for enduring and ameliorating preventive actions aimed at timely HIV testing among all individuals at risk for HIV infection and/or those who present diseases known to be related with HIV infection.

## Abbreviations

CART: Combination antiretroviral therapies
CIs: Confidence intervals
ORs: Odds ratios
IDU: Injecting drug use
ISTAT: Italian National Institute of Statistics
RNAIDS: National AIDS registry
PWA: People with AIDS
